# Review of Bicipital Groove Morphology and Its Analysis in North Indian Population

**DOI:** 10.5402/2013/243780

**Published:** 2013-09-11

**Authors:** Singh Rajani, Singh Man

**Affiliations:** ^1^Department of Anatomy, All India Institute of Medical Sciences, Rishikesh, Uttarakhand 249201, Dehradun UK, India; ^2^DRIAS, 409 Gemscourt Apartment 223 Faizabad Road, Lucknow, Uttar Pradesh 226007, India

## Abstract

The variant morphometry of bicipital groove is reported to be associated with pathologies of biceps tendon and is useful in surgical procedures in this region. The pathologies of biceps tendon are frequent causes of shoulder pain. Therefore, under the condition of paucity of data pertaining to north Indians, not only morphometric analysis of bicipital groove and a new definition of narrow/shallow groove to provide logical explanation for dependence of pathologies of biceps tendon on groove morphology is done but also a review of the literature has been carried out. Various dimensions such as lengths of medial and lateral walls, width, depth, medial wall, and opening angles including incidence of supratubercular ridge of bicipital groove from 101 humerii are 23 ± 5, 32 ± 5, 8 ± 2, 6 ± 1, 48.91 ± 10.31, 82.20 ± 22.62, and 37%, respectively. The average height along with average width of biceps tendon and average width along with average depth of bicipital groove from two cadavers are 1.8, 10.5, 11.3, and 5.5 mm, respectively. The knowledge of bicipital groove will be of paramount importance to anatomists for new data, for orthopaedic surgeons in carrying out surgical procedures in this region, and for physicians in the management of anterior shoulder pain in north Indian population.

## 1. Introduction

Bicipital groove (BG) is an indentation on the anterior aspect of proximal part of humerus. This groove allows tendon of long head of biceps brachi muscle enveloped in synovial sheath and ascending branch of anterior circumflex humeral artery to pass through it. It is bounded medially by lesser tubercle, laterally by greater tubercle, and superiorly by bridging of transverse humeral ligament [1]/muscle fibers of subscapularis, supraspinatus and pectoralis major muscles [2, 3]. This groove with transverse humeral ligament/muscle fibers bridging it provides stability and smooth functioning of tendon of long head of biceps brachi muscle and prevents its subluxation during multidirectional biomechanical movements of arms. Apart from this, the greater function of biceps brachi muscle whose tendon is enshrined in bicipital groove is suppination, flexion, and screwing biomechanical movements. On motion of humerus, the proximal humerus moves in relation to fixed biceps tendon which is firmly held in place at the level of intertubercular sulcus by tuberosities and humeral transverse ligament [4]. With elevation of arm, humerus moves about 3.8 cm on the fixed tendon [5]. In the dynamics of external rotation to internal rotation, the tendon is forced medially against lesser tubercle and superiorly against transverse humeral ligament [6]. Coracohumeral ligament directly overlies the transverse humeral ligament/muscle fibres and is continuous with rotator cuff [7]. Morphometry of BG may influence the functions of surrounding structures leading to various pathological conditions [8, 9]. 

Supratubercular ridge originally described by Meyer in 1928 [5] and later by Hitchcock and Bechtol in 1948 [4] consists of bony protuberance and is continuous with superior aspect of lesser tuberosity. It allows the tendon a more gradual change in direction as it enters the bicipital groove by elevating and forcing it laterally. Thus supratubercular ridge may prevent medial displacement of tendon of the biceps. Incidence of supratubercular ridge has not been studied in North Indian population. Therefore, incidence of spurs and supratubercular ridge in Indian population through this study has also been observed. Apart from this, the bicipital groove is important landmark for replacement of prosthesis of shoulder. Thus knowledge of BG is highly useful in prosthetic sizing, positioning, and designing [10]. Bicipital groove also acts as an important landmark for placement of lateral fin of prosthesis in shoulder arthroplasty and humeral head replacement in fractures of upper end of humerus [11]. In a series of classic reports by various authors, the papers in [4, 12–15] have discussed primary versus secondary biceps tendinitis and different treatment regimens for each of these entities [16, 17]. The association of shoulder pain with pathology of the LHB is currently attributed to inflammation (synovitis), impingement, prerupture, or instability of the tendon at the entry into the bicipital groove (subluxation or dislocation) [16, 18–24]. 

Anterior shoulder pain affects large masses of people including elderly population of the world. Lesions affecting the tendon of LHB brachii have been postulated to be among the most frequent causes of pain and disability in the shoulder. This pain may be caused by rotator cuff, supraspinatus, and biceps tendon diseases [25]. Pathologies of the biceps tendon can be broadly divided into two classes, namely, as follows.

1. Primary tendonitis: Berlemann and Bayley [26] reported the long term results of 14 patients (15 shoulders) following keyhole biceps tenodesis. Fifty-three percent of patients had previously undergone a subacromial decompression but symptoms persisted until the biceps tenodesis was carried out. This would suggest that biceps tendinitis is a primary event.
2. Secondary tendonitis [14, 15, 17, 27]: This may further be subdivided into three main types, inflammatory, instability, and traumatic. Clearly, there is a huge overlap between these categories and in fact biceps pathology is very rarely a single entity [28]. Apart from this, the most recent biomechanical data come from Youm et al. [29] who found that loading of the long head of biceps tendon significantly affects the glenohumeral joint, range of movement, translations, and kinematics. The pathologies as elaborated above change the morphology of BG. Therefore, varied anatomical knowledge of the BG is important as abnormalities of the bicipital tendon and its synovial sheath have been implicated in a variety of causes of shoulder pain and disability [6, 7]. A radiological study recommended that the entire length of the BG be examined to determine the osseous anatomy of the groove [30]. Few authors have studied the morphology of the upper end of the humerus in geographically diversified regions [10, 31–33].

Hence an attempt has been made to examine the length, width, depth, and opening angle of bicipital groove statistically to correlate with clinical implications in north Indian population along with a review of the literature. 

## 2. Materials and Methods

**Figure 1:**
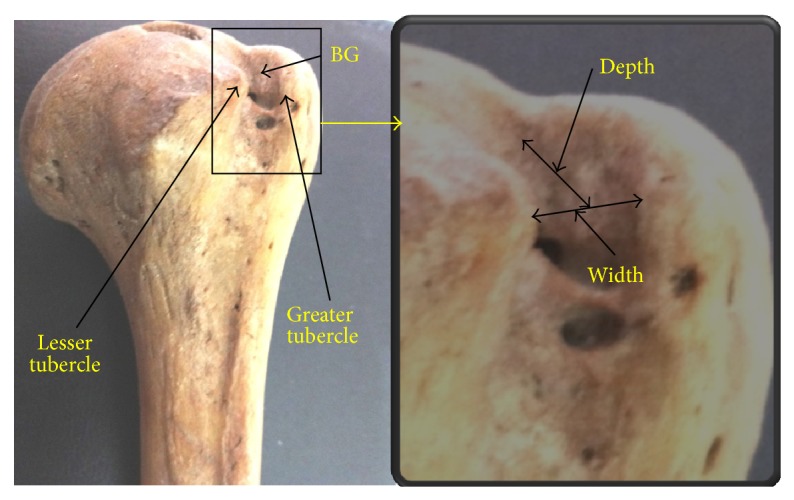
Showing width and depth of BG.

The study was carried out using hundred and one humeri of assorted sex and pair. The subjects consisted of 45 left and 56 right humeri obtained from osteology laboratory of KG Medical University, Lucknow, UP, India. The lengths of medial, lateral walls, depth, lengths, and width of bicipital groove (Figure 1) were measured by digital vernier callipers.

**Figure 2:**
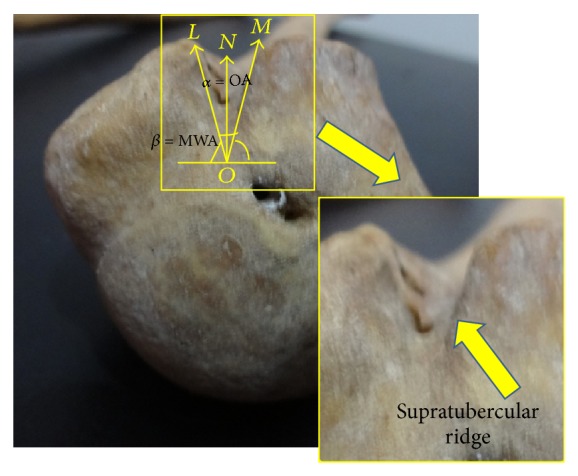
Showing opening (OA), medial wall angle (MWA), and supratubercular ridge.

 The statistical analyses consisting of not only incidences of supratuberecular ridge of Meyer and presence of bony spurs but also mean, standard deviation, range, median and mode of length of medial and lateral walls, width, depth, and opening/medial wall angles of BG were carried out. The opening/medial wall angles of BG (Figure 2) have been computed. 

The narrowness and shallowness of BG have been redefined objectively in relation to dimensions of both bicipital groove and biceps tendon for adequate prediction of pathologies of biceps tendon. Though the precise definition of narrowness/shallowness of BG is difficult yet systematic, relative and constrained definition depending on the dimensions of bicep tendon and its natural abode, that is, bicipital groove, is formulated as follows.

1. If the width of BG is less than the width of biceps tendon, it is a narrow BG which may produce attritional changes causing impingement, inflammation, and degeneration.
2. Similarly, if the depth of the groove is less than height of the bicep tendon, it is shallow causing subluxation or dislocation which in the long run may cause degenerative changes and rupture.

As the new definition of narrowness or shallowness requires the width and height of biceps tendon, so the width and height of 4 biceps tendons from 2 cadavers have also been measured to provide more realistic definition of narrow/shallow of bicipital groove. In addition to this, the review of literature is accommodated in this study.

## 3. Results

The means ± standard deviation of lengths of medial and lateral walls, width, depth and opening/medial wall angles of BG have been computed as shown in Table 1. Mean length of medial wall of BG on right side was 22 ± 4 and that on left side was 23 ± 5 mm (Table 1). Mean length of lateral wall of BG on right side was 31 ± 6 mm and that on left side was 31 ± 5 mm.

**Figure 3:**
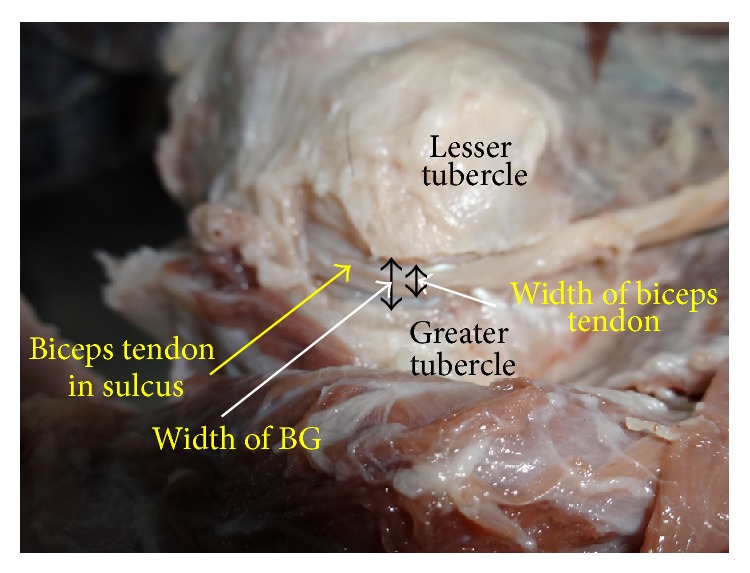
Shows biceps tendon and BG.

Mean width on the superior part of BG on right side was 8 ± 2 mm and that on left side was 8 ± 2 mm. The depth of BG on right side was 5 ± 1 mm and that on left side was 6 ± 1 mm. Average lengths and widths of BG are 80 mm and 12 mm. Average length and width of humerus are 300 mm and 23 mm. The incidence of supratubercular ridge was 37% total, 17% on right side, and 20% on left side. The average length of BG is 26.7% of total length of the humerus and average width of BG is 52% of average width of humerus. The width and height of biceps tendon (Figure 3) have been displayed in Table 2. 

## 4. Discussion

The morphometric study carried out by various authors [25, 34–37] has been compared with present study as depicted in Table 3. 

Lengths of medial wall and lateral walls of BG have not been reported so far except in the present study, so there is no data for comparison. Length of BG in present study is comparable with that of Murlimanju but slightly higher than that observed by Wafae et al. The width of BG under present study is close to that of Cone et al. but slightly higher than that of Murlimanju and lower than that of Wafae et al. Median of width of BG on right and left sides are 8 and 10 mm, respectively, and mode is 8 mm on each side. It indicates that width of BG in most of the north Indian population is 8 mm. In the present study depth of BG more than 3 mm and depth ranging 4–6 mm are 98% and 96% of humeri, respectively, as against 90% and 86% in the study of Cone et al. Median and mode of depth in right/left side are 5/6 mm each. It indicates that groove is deeper on left side. Depth of BG in present study is comparable to Joseph et al. but higher than that of Murlimanju, Cone, and Wafae. Medial wall angle of BG is slightly higher than that of Joseph and lower than Cone et al. Opening angle of BG under present study is very close to that of Joseph. Medial wall angle (MWA) in my study is lower than Cone and higher than Joseph. The median and mode of these morphometric parameters of BG are very useful for prosthetic sizing, positioning, and designing. 

The supratubercular ridge in present study is found in 17% in right and 20% in left totalling to 37% in all humerii. As per Hitchcock and Bechtol [4], there exists a definitive relationship between the presence of supratubercular ridge and tendonitis. Cone et al. [34] from their radiographic interpretations observed this ridge in 50% of cases and reported that it was not pathologically significant. Vettivel et al. [8] observed this ridge in 88% on right side and 57% on left side and emphasised that it was more important on right side than left to prevent medial displacement of long head of biceps from the BG.

In present study mean width and height of biceps tendon (Figure 3) are 10.5 and 1.6 mm, respectively, which is higher than that observed by Lam and Mok [28]. Similarly these dimensions of tendon at the exit from BG are 7 and 1.8 mm, respectively. The width of tendon at the exit is more and height is less than that observed by Lam and Mok [28].

 If the tendon is not encased by median and lateral walls of BG due to its shallow depth it may be dislocated, either partially or fully by biomechanical movements of arms. This dislocation of biceps tendon associated with impingement may cause degeneration leading to partial or full rupture with the passage of time. Apart from this, if the movements of tendon are not free in narrow or in presence of bony spurs in BG during biomechanical movements of shoulder joint, its constant fraying might give rise to pathologies of biceps tendon. Cone et al. [34] reported that wide grooves (i.e., >17 mm) were often shallow. This might predispose to tendon subluxation or dislocation. They could not precisely define the depth at which the tendon became unstable. But in a groove 3 mm deep or less, it should be viewed with suspicion in managing pathologic conditions of the shoulder on patient radiographs as per Cone's view. Pfahler et al. [38] found a flat groove angle associated with radiologic depth less than 2 mm. The pathologic changes involving the biceps tendon were evident on sonography. They found a significant accumulation of pathologically changed biceps tendons when a flat groove angle was present. According to several authors subluxation and dislocation of biceps tendon were more common in presence of shallow bicipital groove [1, 4, 5, 7, 39]. It is also reported that with shallow bicipital groove, the tendon is susceptible to chronic trauma due to impingement by the overlying acromion, rotator cuff, and coracoacromial arch during shoulder movement [13]. A shallow intertubercular groove is vulnerable to impingement damage and subluxation [37, 40]. Rupture of the biceps tendon most commonly occurs proximally near the glenoid labrum and distally in the bicipital groove [28]. Smith [37] designated bicipital groove types as narrow, normal and shallow depending on mean opening angle less than 66°, 94°, and 118°. 

As seen from above description, the objective and realistic definition has not been given by any author. Therefore, present author has attempted reliable, realistic, and objective definition of shallowness of BG in relation to biceps tendon as given in Section 2 of this paper. This definition of shallowness can be realised in situ in live patients only but is expected to elucidate the pathologies associated with shallowness of the groove. As per morphometric data (Table 3) of BG and biceps tendon as observed in two cadavers in the present study, the groove is not shallow according to new definition of shallowness. Figure 3 shows the biceps tendon and BG in a dissected cadaver wherein the biceps tendon is positioned and perfectly well protected in BG. The BG in this case is not shallow as seen in the above-mentioned figure, and the height of biceps tendon is less than depth of BG.

A narrow groove can cause the tendon to develop attritional frictional damage. Continual mechanical stress at anatomically narrow sites (i.e., distal bicipital groove, beneath the acromion or the coracoacromial ligament) and impingement of the biceps tendon in the coracoacromial arch during flexion may cause these well-known degenerative changes [19, 41]. In the present study the data of BG and biceps tendon observed from two cadavers do not indicate narrow BG as per new definition of narrowness and shallowness given in Section 2.

However, in contrast to previous studies, Abboud et al. [25] did not find any conspicuous anatomic findings of the bicipital groove in the shoulders effected by rotator cuff diseases on MRI such as a narrow groove, flat groove, or small medial groove that were predictive of biceps pathology at the time of arthroscopy [37, 38]. This may be because of the following

1. limitations of Josheph's study,
2. subjective/qualitative definition of shallowness and narrowness.

The limitations to Josheph's study were as follows:

1. clearly a selection bias
  - to surgical patients suffering from primary rotator cuff disease,
  - only patients having MRI done,
2. classification of biceps tendon pathology was arbitrary and based purely on visual inspection not on histopathologic changes,
3. MRI is often considered to be less accurate than X-ray or computed tomography scan in evaluating bony dimensions.

## 5. Clinical Significance of BG Morphology

Lesions due to pathology of biceps tendon have been postulated to be among the most frequent causes of pain and disability in the shoulder. Biceps tendon pathology has been visualized in three main categories, namely, instability, inflammatory, and traumatic [28]. Abboud et al. [25] divided the biceps tendon pathology in normal, inflamed, partially torn, or ruptured tendon. Acute inflammatory and chronic degenerative alterations causing partial/complete rupture and subluxation/dislocation can be found in the long head of the biceps tendon [4]. Instability of biceps tendon besides other factors may be attributed to length of medial/lateral walls, opening/medial wall angles depending on width/depth constituting shallowness of BG, and presence of supratubercular ridge [37, 40, 41]. The implication of longer walls is expected to ensure greater stability to biceps tendon lying in the bicipital groove than the shorter walls during multidirectional biomechanical movements. But the rider to this fact is that it may also cause attritional friction in a longer length of biceps tendon surrounded by longer walls creating inflammation under narrow conditions of BG. As the lengths of medial and lateral walls decrease, the instability increases and the tendon is likely to be damaged. The inference drawn is based on reconstruction of anatomical model of this part of the human body advancing the knowledge and experience of anatomy and clinical studies recorded in the literature supported by logical force as the study is on dry bones. Range provides an idea of length of these walls in north Indian population, whereas the mean ± SD reveals the average size of BG. The median may be very useful in planning surgical procedures in this part of the body. Mode is representative of most frequent incidence of lengths of these walls in the subject population. If the instability of biceps tendon is studied in relation to lengths of BG most frequent value of length of walls may play a vital role in diagnostics of tendon instability or attritional damage. 

Cone et al. felt that a groove 3 mm deep or less and more than 17 mm wide may predispose to tendon subluxation or dislocation on patient radiographs. The flat groove of Pfahler et al. [38] was found to depict significant accumulation of pathologic changes in biceps tendon in 62% of cases on sonography. The supratubercular ridge of Meyer and a prematurely shallow bicipital or intertubercular sulcus have been postulated to result in a variety of lesions after repetitive use or acute trauma [37, 40, 41]. These include acute or chronic peritendonitis, varying degrees of attrition or damage to the tendon, and subluxation or complete dislocation.

As the biceps tendon is enshrined in BG, width may influence the pathology occurring in this tendon. In wider groove the tendon is more free to move and there are less chances of tendon getting damaged.

## 6. Conclusions

- The morphometry of the bicipital groove in terms of length of media wall, lateral wall, length of BG, width, depth medial wall angle, and opening angle has been elucidated with reference to north Indian population.
- The data on morphometry of BG will be of utmost use for anatomist, radiologists, orthopaedic surgeons, and physicians.
- The new definition of narrow/shallow BG has been given.

## Figures and Tables

**Table 1:** Morphometric measurements of BG.

Parameters	Mean ± SD in mm			Range in mm		
	Right	Left	Total	Right	Left	Total
MWL	22 ± 4	23 ± 5	23 ± 5	14–34	12–32	12–34
LWL	31 ± 6	31 ± 5	32 ± 5	15–45	19–44	15–45
WS	08 ± 02	08 ± 02	08 ± 2	04–18	04–12	04–18
D	5 ± 1	6 ± 1	6 ± 1	3–9	3–10	3–10
MWA (°)	47.34 ± 9.61	50.85 ± 10.93	48.91 ± 10.31	25.48–67.44	32.68–69.53	25.48–69.53
OA (°)	85.3 ± 19.2	78.31 ± 21.85	82.20 ± 20.62	49.14–129.04	40.95–114.66	40.95–129.04

**Table 2:** Morphometry of biceps tendon.

Biceps tendon parameters		Right shoulder (measurement in mm)		Leftt shoulder (measurement in mm)	
Cadaver	Parameters	At entry in BG	At exit in BG	At entry in BG	At exit in BG
Cadaver-1	Width of tendon	5	6	6	6
	Height of tendon	1	1	2	2
	Width of BG	8	—	6	—
	Depth of BG	6	—	6	—

Cadaver-2	Width of tendon	15	6	16	10
	Height of tendon	2	2	2	2
	Width of BG	15		8	
	Depth of BG	7		3∗	

^*^1.5 mm bony growth + 1.5 clear depth of BG.

**Table 3:** Comparison of length, width, and depth of bicipital groove.

Study	Wafae et al. [36]	Cone et al. [34]	Abboud et al. [25]	Murlimanju et al. [35]		Present	
Parameters				R	L	R	L
Length	81	NA	NA	86 ± 10.1	83.3 ± 11.5	85 ± 0.9	83 ± 10.1
Width	10.1	8.8	NA	8.3 ± 2.4	8.7 ± 2.2	9.0 ± 2.1	8.9 ± 1.1
Depth	4	4.3	5.1	4.7 ± 2.0	4.2 ± 1.6	5.0 ± 1.0	6.0 ± 1.0
Median wall angle	NA	56	47	NA	NA	47.34 ± 9.61	50.85 ± 10.93
Opening angle	NA	NA	81	NA	NA	85.3 ± 19.2	78.31 ± 21.85
